# Post-surgical inflammatory neuropathy after anterior cruciate ligament repair: a case report

**DOI:** 10.1186/s13741-024-00384-w

**Published:** 2024-04-02

**Authors:** Lisa Y. Sun, Andrew T. Gray, Matthias R. Braehler

**Affiliations:** 1 Department of Anesthesiology, University of Minnesota, 420 Delaware St. SE, Minneapolis, MN 55455 USA; 2 Department of Anesthesia and Perioperative Care, Zuckerberg San Francisco General Hospital and Trauma Center, University of California, San Francisco, CA 94110 USA; 3 Department of Anesthesia and Perioperative Care, University of California San Francisco, 521 Parnassus Avenue, San Francisco, CA 94143 USA

**Keywords:** Peripheral neuropathy, Inflammatory neuropathy, Post-surgical, Peripheral nerve block

## Abstract

**Background:**

Unanticipated symptoms of peripheral nerve damage following surgery are distressing to both the patient and their clinical team, including surgeons, anesthesiologists, and neurologists. The causes that are commonly considered for perioperative neuropathy can include surgical trauma, positioning-related injury, or injury related to a regional anesthetic technique. However, these cases often do not have a clear etiology and can occur without any apparent periprocedural anomalies. Postoperative inflammatory neuropathy is a more recently described, and potentially underrecognized cause of perioperative neuropathy which may improve with corticosteroid therapy. Therefore, it is an important etiology to consider early in the evaluation of perioperative neuropathy.

**Case presentation:**

An otherwise healthy patient presented for left anterior cruciate ligament reconstruction. He underwent femoral and sciatic ultrasound-guided single-injection peripheral nerve blocks preoperatively, followed by a general anesthetic for the surgical procedure. He developed postoperative neuropathy in the sciatic distribution with both sensory and motor deficits. The patient received multi-disciplinary consultations, including neurology and pain management, and a broad differential diagnosis was considered. Based on neurological evaluation and imaging studies, a final diagnosis of post-surgical inflammatory neuropathy was made. The patient’s course improved with conservative management, but immunosuppressive treatment may have been considered for a more severe or worsening clinical course.

**Conclusions:**

There are limited publications describing postoperative inflammatory neuropathy, and this case serves to illustrate a potentially under-recognized and multifactorial cause of postoperative neuropathy. Perioperative neuropathies are a complication that surgeons and anesthesiologists strive to avoid; however, prevention and treatment of this condition have been elusive. Increased reporting and investigation of postoperative inflammatory neuropathy as one cause for this complication will help to further our understanding of this potentially devastating complication.

## Background

Post-surgical neuropathies are usually attributed to mechanical injuries (such as nerve compression, stretch, contusion, or transection), local anesthetic toxicity, and ischemia. However, they can also occur in the setting of post-surgical nerve inflammation, which has only recently been better characterized in the literature (Staff NP et al. 2010) and can present as focal, multifocal, or diffuse neuropathy. These inflammatory neuropathies can occur temporally and spatially distant from the surgical time and site. There are a limited number of case reports of post-surgical inflammatory neuropathies, all with varying presentations (Staff NP et al. 2010; Rattananan et al. 2014; Ahn et al. 2011; Cetiz 2017; Warner and Warner 2014; Laughlin et al. 2014). Our case demonstrates the utility of a thorough evaluation of postoperative neuropathy, which resulted in the diagnosis of post-surgical inflammatory neuropathy.

## Case presentation

**Fig. 1:**
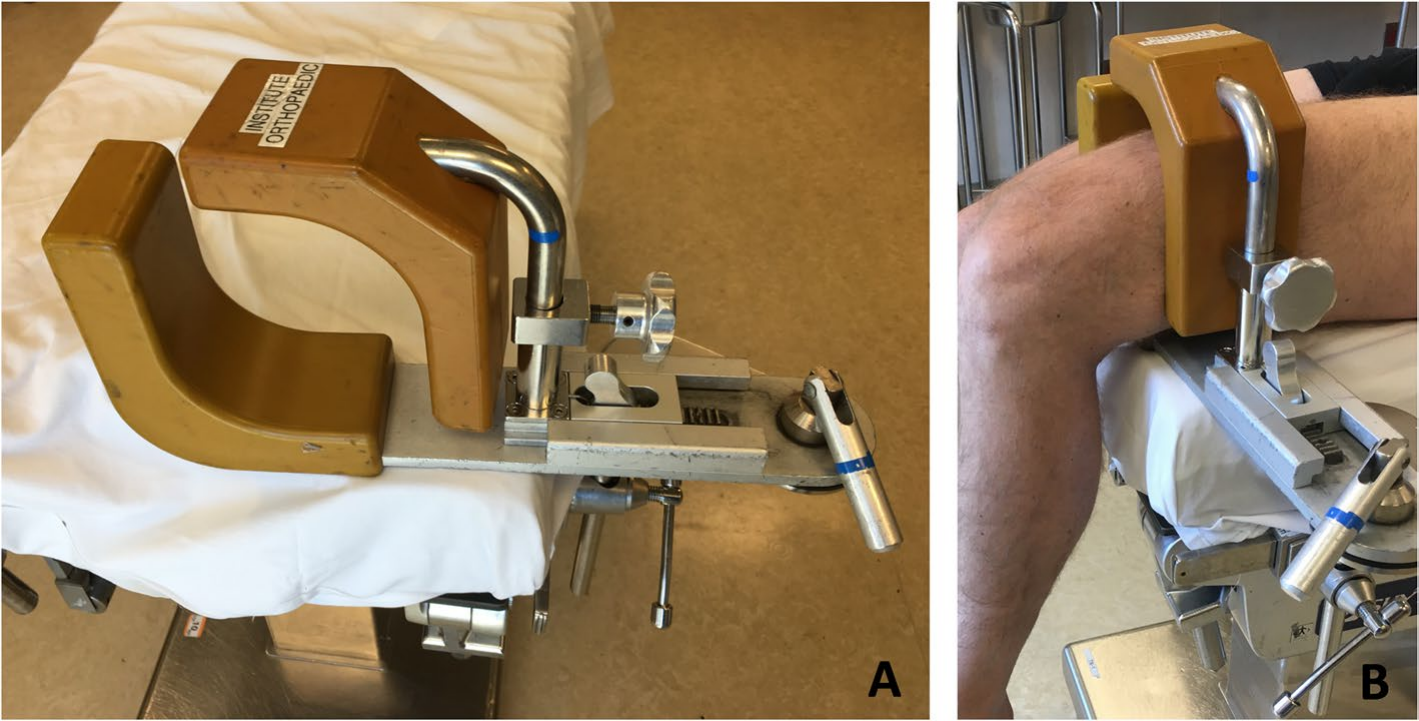
**A** Thigh holder. **B** Depiction of leg position within thigh holder. The padding and thigh tourniquet used on an actual patient’s leg in a thigh holder is not shown

A 25-year-old, 99 kg, otherwise healthy male presented for left anterior cruciate ligament reconstruction with patellar tendon autograft. The patient received femoral and sciatic ultrasound-guided peripheral nerve blocks with 0.5% ropivacaine (25 ml per site). The blocks were uneventful, with no paresthesia and no high pressure noted during manual injection. The patient received general anesthesia with a supraglottic airway. The patient was positioned supine with the operative leg slightly elevated in a thigh holder and flexed 90° at the knee (Fig. 1). The tourniquet was inflated to 250 mmHg for 32 min. No posterior work, such as a hamstring harvest, was performed.

The patient developed shock-like pain, tingling, and numbness in the plantar aspect of his foot as well as numbness along the left lateral leg on postoperative day (POD) 2, with no motor deficits on exam. His neuropathic pain worsened over the following weeks. He did not endorse systemic inflammatory symptoms, such as fevers or chills. Neurologic consultation on POD 23 identified decreased sensation to light touch, vibration, and pinprick in the sciatic territory; reduced toe flexion and extension (3/5 according to the Medical Research Council scale); reduced foot dorsiflexion and plantar flexion (4/5); and absent left Achilles reflex. Electromyography showed decreased motor nerve conduction responses for the left peroneal and tibial nerves and absent responses for the left sural and superficial peroneal nerves. These results suggested a sciatic neuropathy, without evidence of radiculopathy, likely due to perioperative inflammatory sciatic neuritis. Conservative management and close follow-up were recommended.

**Fig. 2:**
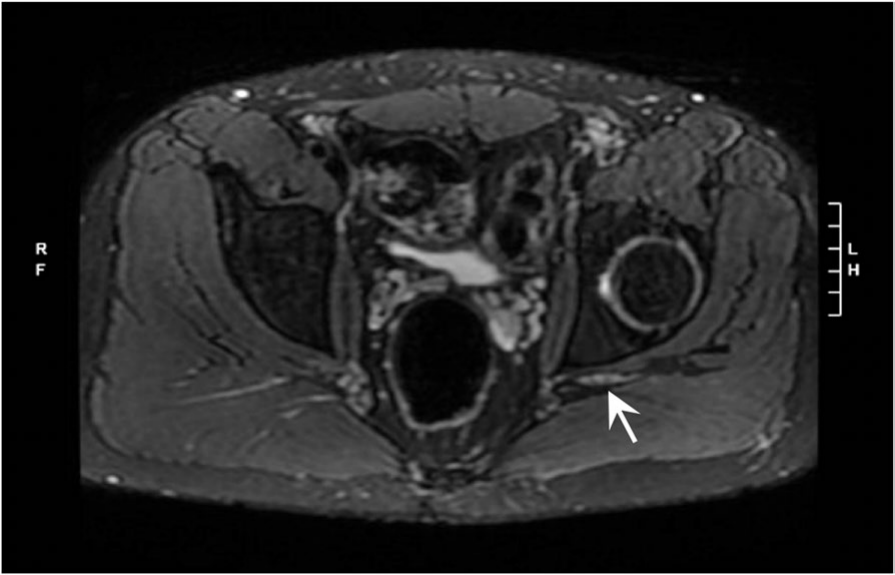
Magnetic resonance neurogram showing asymmetric T2 hyperintensity and enlargement of the left sciatic nerve

The patient was also seen by pain management specialists, and other possible diagnoses were considered, including radiculopathy and complex regional pain syndrome. Magnetic resonance (MR) imaging of the lumbar spine on POD 42 showed left L5-S1 disk extrusion with nerve compression but left L5, S1 foraminal steroid epidural injections were performed without relief. The patient’s hyperesthesia led to the consideration of complex regional pain syndrome, although no vasomotor or sudomotor symptoms were noted. On POD 52, a lumbar sympathetic nerve block (0.25% bupivacaine) along with a distal ankle block (1% lidocaine with 0.375% bupivacaine) were performed, and they provided significant pain relief. Ongoing diagnostic uncertainty prompted MR neurogram from the lumbar spine to the foot on POD 67, which showed neuritis in the sciatic nerve distribution starting in the pelvis (above the level of surgery, thigh holder, tourniquet, and nerve blocks). This was consistent with a diagnosis of post-surgical inflammatory neuropathy (Fig. 2). At this time, the patient was experiencing improved pain control after adjustment of a variety of neuropathic and narcotic pain medications over his postoperative course. He also had improved function and weight-bearing on the operative extremity with ongoing physical therapy. On 1-year follow-up, the patient had significant improvement with near resolution of symptoms, with a pain regimen consisting of duloxetine and diclofenac.

## Discussion

This case report describes the clinical course, evaluation, and diagnosis of post-surgical inflammatory sciatic neuropathy, in a patient without any known risk factors. As is common in the setting of postoperative neuropathy, this patient experienced evolving symptoms, multi-disciplinary consultations, a broad differential diagnosis, and a variety of therapies for pain management. His final diagnosis of post-surgical inflammatory neuropathy was ultimately supported by imaging studies and the exclusion of other etiologies, as there are no known markers for this condition. His course improved with conservative management, but consideration for immunosuppressive treatment may have been given to a more severe or worsening clinical course.

The incidence of post-surgical inflammatory neuropathy is unclear, and it may be under-appreciated (Staff NP et al. 2010). The cause of this condition is likely multifactorial, and given its heterogeneous presentation in prior case reports, it may encompass multiple different entities. It is thought to be an immunological response to stress, such as surgery. Based on a retrospective study (Staff NP et al. 2010), additional possible risk factors may include diabetes mellitus, tobacco use, cancer, and infection; in this case, the patient did not have any of these suggested risk factors. Given the rarity of this condition, more case reports and studies would be useful to further elucidate predisposing factors. This condition should be included in the differential diagnosis for post-surgical neuropathy, particularly when there is no clear mechanical insult to explain the neuropathy.

**Fig. 3:**
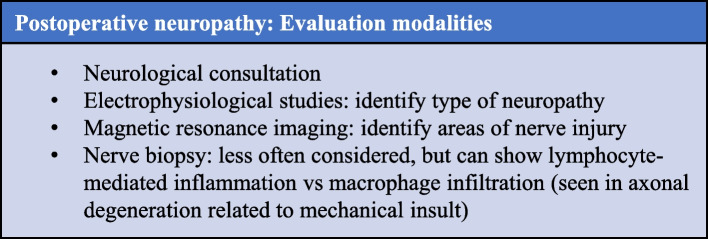
Evaluation modalities for postoperative neuropathy

Evaluation of postoperative nerve injury includes neurological consultation, as well as consideration of ancillary testing (Fig. 3). Electrophysiological studies can be useful for defining the type of neuropathy. MR imaging can help to identify areas of nerve injury. Nerve biopsy is less often considered, but it can show lymphocyte-mediated inflammation (as opposed to macrophage infiltration, which is seen in axonal degeneration related to mechanical insult) (Staff NP et al. 2010; Rattananan et al. 2014; Ahn et al. 2011).

The optimal treatment and management of inflammatory neuropathy is yet to be determined. Some cases have improved with conservative management, as with this patient. Other cases of inflammatory neuropathy have improved with immunotherapy, including intravenous immunoglobulin and steroids (such as methylprednisolone and prednisone) (Staff NP et al. 2010; Rattananan et al. 2014; Ahn et al. 2011; Cetiz 2017; Warner and Warner 2014; Laughlin et al. 2014). Although this treatment has only been reported in retrospective studies and case reports, it does suggest that early consideration and prompt diagnosis of this entity is important so that suppression of the immune response can be considered. For this reason, it is important for anesthesiologists and surgeons to be aware of this condition when confronted with a case of post-surgical neuropathy. Future prospective studies would be helpful to demonstrate the true incidence of post-surgical inflammatory neuropathy, predisposing risk factors, effectiveness of immunotherapy, and prophylactic role of steroids pre- or intra-operatively.

## Data Availability

Not applicable.

## Abbreviations

POD: Postoperative day
MR: Magnetic resonance

## References

[CR1] Ahn KS, Kopp SL, Watson JC, Scott KP, Trousdale RT, Hebl JR (2011). Postsurgical inflammatory neuropathy. Reg Anesth Pain Med..

[CR2] Cetiz A (2017). Post-surgical focal inflammatory neuropathy of the sciatic nerve. Acta Neurol Belg..

[CR3] Laughlin RS, Dyck PJ, Watson JC (2014). Ipsilateral inflammatory neuropathy after hip surgery. Mayo Clin Proc..

[CR4] Rattananan W, Thaisetthawatkul P, Dyck PJ (2014). Postsurgical inflammatory neuropathy: a report of five cases. J Neurol Sci..

[CR5] Engelstad J, Klein CJ, Amrami KK, Spinner RJ, Dyck PJ, Staff NP (2010). Post-surgical inflammatory neuropathy. Brain..

[CR6] Warner ME, Warner MA (2014). Inflammatory neuropathy: a potentially treatable etiology for a subset of perioperative neuropathies. Mayo Clin Proc..

